# Preclinical development of the quadrivalent meningococcal (ACYW) tetanus toxoid conjugate vaccine, MenQuadfi®

**DOI:** 10.1007/s10719-022-10050-2

**Published:** 2022-04-20

**Authors:** Richard Kensinger, Arun B. Arunachalam

**Affiliations:** 1 BioProcess R&D, Sanofi Pasteur, 1 Discovery Dr, Swiftwater, PA 18370 USA; 2 Analytical Sciences, R&D Sanofi Pasteur, 1 Discovery Dr, PA 18370 Swiftwater, USA

**Keywords:** Carrier protein, Glycoprotein conjugate vaccine, Immunogenicity, Meningococcal, Polysaccharide, Vaccine development

## Abstract

Bacterial capsular polysaccharide vaccines are generally poorly immunogenic in infants and older adults. The immunogenicity of capsular polysaccharide vaccines can be improved by conjugating them to immunogenic carrier proteins. One of the most recently licensed conjugate vaccines is the quadrivalent meningococcal vaccine with serogroups A, C, Y, and W conjugated to a tetanus toxoid protein carrier (MenACYW-TT; MenQuadfi, Sanofi Pasteur, Swiftwater, PA, USA). MenACYW-TT was developed to induce optimal immune responses against each of the meningococcal serogroups A, C, W, and Y, and across all age groups, especially infants and older adults (those aged ≥ 50 years). Here, we detail the early iterative vaccine development approach taken, whereby many different ‘small-scale’ conjugate vaccine candidates were prepared and examined for immunogenicity in a mouse model to identify the most immunogenic vaccine. Additional insights from phase I clinical studies informed further optimization of the vaccine candidates by tailoring their conjugation parameter attributes for the optimal immune response in humans. The parameters studied included: different carrier proteins [PR]; polysaccharide [PS] sizes; conjugation chemistries [linker vs. no-linker; lattice vs. neoglycoprotein; activation/derivatization levels]; conjugate size; PS:PR loading ratio; percent free PS; percent free PR; and *O*-acetylation content. The lead quadrivalent conjugate vaccine (polysaccharides of > 50 kDa size conjugated to TT at a high PS:PR ratio via reductive amination for serogroups C, W and Y, and carbonyldiimidazole/adipic acid dihydrazide linker chemistry for serogroup A) empirically identified from the extensive preclinical studies, was ultimately confirmed by the robust antibody responses observed in all age groups in the various clinical studies, including in the most challenging infant and older adult age groups, and subsequently led to the licensed formulation.

**Graphical abstract:**

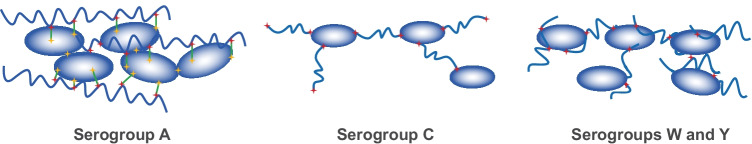

**Supplementary information:**

The online version contains supplementary material available at 10.1007/s10719-022-10050-2.

## Introduction

Invasive meningococcal disease, which typically manifests as meningitis or septicemia, is a highly contagious illness caused by the Gram-negative bacterium, *Neisseria meningitidis*. Twelve meningococcus serogroups have been identified based on their capsular polysaccharides, of which serogroups A, B, C, W, X and Y cause nearly all clinical cases worldwide, with great variation in serogroup by geographic region [1, 2]. The disease remains a global public health concern, due to its potential for causing epidemic outbreaks with associated high morbidity and mortality. The disease burden varies substantially with the highest rates observed in ‘meningitis belt’ countries in northern Africa caused by serogroup A, where large epidemics with incidence of 100–800 per 100,000 people occurred every 5–12 years [3] prior to the introduction of the monovalent conjugate MenA vaccine (MenAfriVac). Meningococcal disease can have devastating sequelae that can adversely impact the quality of life of survivors in both the short- and long-term, and has a 8–15% case fatality rate despite “state-of-the-art medical care” (50–80% case fatality rate when untreated) [2, 4, 5]. Although all age groups are at risk of meningococcal disease, the highest disease burden is in infants and children aged < 5 years, adolescents/young adults and the elderly (aged ≥ 65 years) [6, 7].

Meningococcal vaccines have been pivotal in the prevention of meningococcal disease, with polysaccharide-conjugate vaccines preferred over unconjugated-polysaccharide vaccines due to their increased immunogenicity in all age groups, longer persistence of immunity, booster effect, ability to reduce meningococcal nasopharyngeal carriage (though this has recently be questioned [8]) and their potential for herd protection [2, 9, 10]. There are a number of mono- or multivalent meningococcal conjugate vaccines available including combination conjugate vaccines [2, 11]. Four quadrivalent meningococcal conjugate vaccines are currently available in the global market; all of these contain serogroups A, C, W, and Y capsular polysaccharides, but differ in a number of attributes including the type of carrier protein used (diphtheria toxoid [DT]; non-toxic mutant of diphtheria toxin, cross reacting material 197 [CRM_197_]; or tetanus toxoid [TT]) [11], conjugation chemistry and their presentation (liquid formulation, or lyophilized with diluent for reconstitution). All four vaccines provide protective antibodies against meningococcal A, C, W and Y disease, although induced immune responses to the different serogroups vary to some extent between vaccines and there are differences in the dosing schedules and the licensed ages for use.

The latest quadrivalent meningococcal conjugate vaccine to become available, MenACYW-TT (MenQuadfi, Sanofi Pasteur, Swiftwater, PA, USA), was developed to induce optimal immune responses against each serogroup (A, C, W, and Y), for all age groups, including infants and the elderly. Early vaccine development took an iterative approach, whereby many different ‘small-scale’ conjugate vaccine candidates were prepared and examined for immunogenicity in a mouse model to identify the most immunogenic vaccine. The conjugate candidates were subsequently refined as additional insights from phase I clinical studies became available to further optimize and tailor their conjugation parameter attributes for the optimal immune response in humans. The parameters studied included: different carrier proteins [PR]; polysaccharide [PS] sizes; conjugation chemistries [linker vs. no-linker; lattice vs. neoglycoprotein; activation/derivatization levels]; conjugate size; PS:PR loading ratio; percentage of free PS; percentage of free PR; and *O*-acetylation content. Here, we present the results of the preclinical animal studies that helped define the ranges of the parameters for the manufacturing of MenACYW-TT vaccine batches under current good manufacturing practice (cGMP) conditions for the clinical trials, and that subsequently led to the licensed formulation.

## Methods

### Ethics statement

All studies with the mice were approved by the Institutional Animal Care and Use Committee (IACUC) and undertaken in accordance with the recommendations in ‘The Guide for the Care and Use of Laboratory Animals’ published by the National Research Council (Washington DC) [12].

### Mouse immunogenicity model

A mouse immunogenicity model optimized for assessing the immunogenicity of meningococcal polysaccharide conjugate vaccines was used for identifying an optimal MenACYW polysaccharide-conjugate formulation [13]. In brief, groups of 10 female Institute for Cancer Research (ICR) mice aged 6–8 weeks were injected subcutaneously on day 0 and day 14 (or 15) with 0.25 mL of candidate vaccine conjugate formulations according to the study objectives as detailed in the next section. The vaccine preparations were tested at a dosage of 0.25 µg of each meningococcal serogroup polysaccharide/dose. Each study included a negative control group that received saline buffer instead of vaccine on days 0 and 14. The mice were sacrificed on day 28 (14 days after the second injection) and their blood was collected for serogroup-specific anti-polysaccharide antibody analysis.

### Vaccine preparations

The initial series of studies assessed the impact of the carrier protein, conjugation chemistry, size and nature of the polysaccharide on the immunogenicity of the conjugate vaccine using meningococcal serogroup C polysaccharide conjugate preparations. Initially, serogroup C was chosen as a model serogroup to accommodate assessment of multiple conjugation parameters because substantial information regarding monovalent serogroup C vaccines existed at the time of these initial studies (early 2000’s). Meningococcal serogroup C polysaccharides with sizes of 10 kDa, 20 kDa, or 50 kDa as *O*-acetylated or partially de-*O*-acetylated polysaccharides were conjugated to DT, TT, or recombinant exoprotein A of *Pseudomonas aeruginosa* (rEPA) using different conjugation chemistries (CRM_197_ was not available at that time of these initial assessments). The conjugation chemistries used are summarized in Fig. S1 and are described in detail in Hermanson [14] and included conjugation of the depolymerized polysaccharide (depolymerized with either sodium [meta] periodate or hydrogen peroxide) to the specific carrier protein by: reductive amination; amine coupling using 1-ethyl-3- (3 dimethylaminopropyl)-carbodiimide (EDAC) through an end-linked adipic acid dihydrazide (ADH) linker (ADH/EDAC conjugation chemistry); reductive amination of sodium metaperiodate activated polysaccharide to ADH-derivatized rEPA (ADH-rEPA) carrier protein; and lastly brominated (Br) carrier proteins conjugated to a cystamine (Cys-) derivatized polysaccharide via a thioether linker (Br-Cys) [14]. The physicochemical characteristics of the monovalent serogroup C conjugates formulated for assessment are summarized in Table S1.

Assessment of the polysaccharide loading ratio (weight:weight ratio of the total polysaccharide [PS] divided by the total protein [PR]; PS:PR loading ratio) that generates an optimal immune response was conducted using meningococcal (serogroups A, C, W, and Y) polysaccharide conjugate formulations prepared with three different carrier proteins (TT, rEPA, or CRM_197_ when it became available; DT was subsequently de-prioritized). The polysaccharide loading ratios were varied by changing the conjugation stoichiometries of the polysaccharides and proteins in the conjugation reactions. Monovalent conjugates were designated in general as being ‘low’ (~ 0.3 to ~ 0.5), ‘mid’ (~ 0.5 to ~ 0.7) or ‘high’ (~ 0.7 or higher) loading ratios. The monovalent conjugates were then formulated into tetravalent formulations generally corresponding to ‘low’, ‘mid’, or ‘high’ PS:PR loading ratios (Table S2).

The *O*-acetylation status of the tetravalent (A, C, W and Y) polysaccharide conjugate formulations was also assessed to provide additional insights into the optimal vaccine formulation. The tetravalent (ACWY) polysaccharide conjugate formulations were prepared from monovalent conjugates that were produced by reductive amination on TT and rEPA. After conjugation by reductive amination, C, W, and Y monovalent conjugates were partially de-*O*-acetylated by base treatment as described by Hermanson [14]. The polysaccharide conjugate formulations assessed for each carrier protein included: *O*-acetylated serogroups A, C, W, and Y; and *O*-acetylated serogroup A with de-*O*-acetylated serogroups CWY.

The immunogenicity of different serogroup A monovalent conjugates prepared with different conjugation chemistries (with and without an ADH linker), were compared when formulated with the phase I cGMP clinical batches of monovalent conjugates C, W, and Y conjugated to either TT or rEPA. The serogroup A conjugates compared were: 1) carbonyldiimidazole (CDI-) activated native serogroup A polysaccharide conjugated directly to TT or rEPA; 2) CDI-activated and ADH-derivatized native serogroup A polysaccharide, conjugated to TT or rEPA via amine coupling using EDAC; and 3) a slightly modified ADH/EDAC conjugation chemistry (peroxide depolymerized serogroup A polysaccharide down to ~ 40 kDa, end-derivatized with ADH, purified by diafiltration, and then conjugated to TT or rEPA via amine coupling using EDAC). The physicochemical characteristics of the monovalent conjugates that were reformulated into tetravalent formulations for immunogenicity assessment are summarized in Table S3.

The immunogenicity of developmental serogroup A-TT and -rEPA conjugates prepared using CDI-ADH/EDAC conjugation chemistry was also compared with that of the CDI serogroup A-TT conjugates with no ADH linker. The different conjugates were each tested as tetravalent formulations. The immunogenicity associated with increasing amounts of ADH linkers added to the serogroup A CDI-activated polysaccharide, prior to conjugating to either TT or rEPA via amine coupling using EDAC, was also assessed. A series of TT and rEPA conjugates were prepared from different lots of CDI-ADH-derivatized serogroup A polysaccharides with increasing amounts of ADH derivatization on the serogroup A polysaccharides. The amount of ADH linker added per serogroup A polysaccharide was defined as a mole ratio of polysaccharide repeat units (RU) per ADH linker, where the higher the number of polysaccharide RU units per ADH linker meant fewer ADH linkers were added per polysaccharide chain. The physicochemical characteristics of the monovalent conjugates that were reformulated into tetravalent formulations for immunogenicity assessments are summarized in Table S4.

### Serological assessments

Individual sera from each group were tested by enzyme-linked immunosorbent assay (ELISA) for total polysaccharide-specific IgG antibody levels, and pooled sera were tested for functional antibodies by serum bactericidal assay (SBA). SBA testing of individual sera could not be performed due to limited sera volumes; sub-pools were prepared by combining two mouse sera from the same group to obtain five sub-pools per group (or all sera were pooled into one pool and tested). ELISA and SBA were performed as described in detail elsewhere [13]. In brief, ELISA plates were coated with meningococcal polysaccharide in the presence of methylated serum albumin and the blocked plates were incubated with serially diluted test sera, reference standard and control sera for 90–120 min at 37 °C. Bound antibodies were detected using an anti-mouse IgG enzyme conjugate and substrate combination. The intensity of color was read and specific antibody concentrations in sera were calculated against reference standards. Antibody titers were expressed in mouse ELISA units (MEU) or µg/mL. The SBA was performed using meningococcal reference strains serogroups A (F8238), C (C-11), W (2515) and Y (3021) obtained from the Centers for Disease Control and Prevention, Atlanta, GA, USA. Bacteria in their log phase of growth were incubated with serially diluted sera and baby rabbit complement, and bacterial colonies were counted following incubation overnight at 37 °C. Reciprocal serum dilution yielding ≥ 50% bacterial killing was expressed as the functional antibody titer.

### Statistical analysis

Descriptive statistics were reported for all immunogenicity variables; serogroup-specific anti-polysaccharide IgG and bactericidal antibody responses were described using geometric mean concentrations (GMCs) and geometric mean titers (GMTs), respectively. For IgG GMCs, bootstrapped 95% confidence intervals (CIs) were calculated in R open source statistical software version 4.0.2 using the accelerated bias-corrected percentile limits method [15]. The 95% CIs could not be ascertained for the SBA data since sample size was limited in most cases (samples pooled due to insufficient volumes of sera for the assessment). To evaluate the effect of specific conjugate characteristics on the immunogenicity, conjugate formulations that differed in only one primary characteristic (i.e. carrier protein, polysaccharide size, conjugation chemistry, *O*-acetylation status) were grouped and the results compared.

## Results

### Monovalent serogroup C polysaccharide conjugate

#### Comparison of carrier proteins

**Fig. 1:**
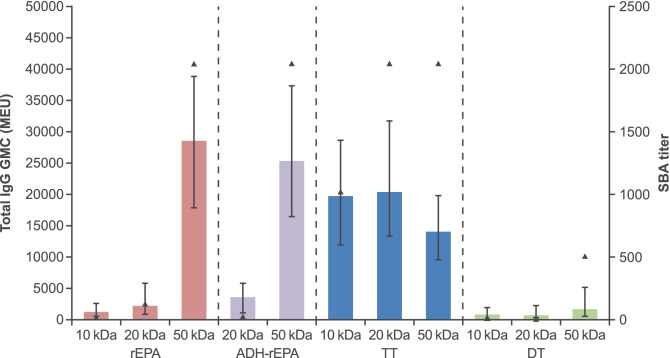
Effect of polysaccharide size on serogroup C specific antibody responses induced with 0.25 µg monovalent dose of serogroup C conjugates prepared with rEPA, TT and DT carrier proteins (NaIO_4_ depolymerized serogroup C polysaccharide [to required molecular weight] conjugated to carrier protein by reductive amination or ADH-derivatized rEPA). Bars represent total IgG level (GMCs with 95% CIs) and triangles represent SBA titer (all sera were pooled into one pool and tested). MEU; mouse ELISA units

**Fig. 2:**
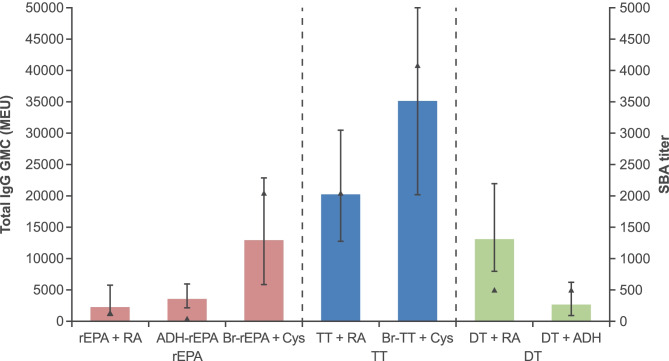
Effect of conjugation chemistry on serogroup C specific bactericidal antibody responses induced with 0.25 µg monovalent dose of serogroup C (20 kDa molecular weight polysaccharide) conjugates prepared with rEPA, TT and DT carrier proteins (RA, reductive amination; ADH, reductive amination to adipic acid dihydrazide-derivatized carrier protein; Br-Cys, brominated carrier proteins conjugated to a cystamine-derivatized polysaccharide via a thioether linker). Bars represent total IgG level (GMCs with 95% CIs) and triangles represent SBA titer (all sera were pooled into one pool and tested)

**Fig. 3:**
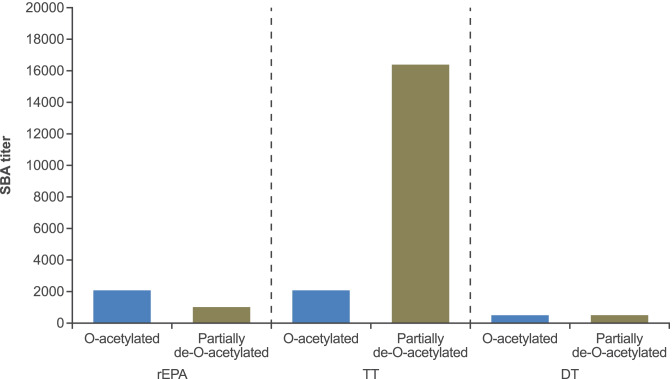
Immunogenicity of *O*-acetylated and partially de-*O*-acetylated 0.25 µg monovalent dose of serogroup C conjugates prepared with rEPA, TT and DT carrier proteins (50 kDa molecular weight polysaccharide via reductive amination) (all sera were pooled into one pool and tested)

All monovalent serogroup C polysaccharide conjugated formulations, irrespective of carrier protein (initially those conjugated to TT, rEPA or DT were assessed), generated polysaccharide-specific IgG and bactericidal antibodies. Mice immunized with formulations prepared with TT, however, regardless of polysaccharide size, PS:PR loading ratio (range 0.11–0.52; Table S1), conjugation chemistry or *O*-acetyl content generally had higher total IgG and bactericidal antibody responses than those immunized with conjugate formulations prepared with either rEPA or DT (see below; Figs. 1–3).

#### Effect of polysaccharide size

Polysaccharide size appears to influence serogroup C-specific antibody responses (total IgG and bactericidal) (Fig. 1) when conjugated to rEPA or DT (to a lesser extent). In general, polysaccharide-specific antibody responses were higher (or highest) for conjugate formulations prepared with the 50 kDa polysaccharide. This was also the case when comparing 20 kDa vs 50 kDa polysaccharide conjugated to ADH-derivatized rEPA (Fig. 1). For the TT conjugates, polysaccharide size did not appear to influence the immune response markedly, considering both serogroup C-specific total IgG and bactericidal antibody titers. Responses to DT conjugates were generally low irrespective of polysaccharide size.

#### Effect of conjugation chemistry

The use of an ADH linker did not make a meaningful difference to the polysaccharide-specific total IgG and bactericidal antibody responses (conjugates prepared with 20 kDa polysaccharide are shown as examples in Fig. 2). Although conjugates prepared with Br-Cys conjugation chemistry appeared to generate higher responses than conjugates prepared using other conjugation chemistries, it was deprioritized for two reasons: 1) preference for large lattice conjugates for improved stability and potentially greater immunogenicity in infants, and 2) ease of GMP manufacturing (minimizing the number of process steps). Therefore, the early process development focused on reductive amination conjugation chemistry.

#### Effect of partial de-O-acetylation

Partial de-*O*-acetylation of serogroup C polysaccharide prepared with TT markedly increased bactericidal titers compared to the control conjugate prepared with *O*-acetylated serogroup C polysaccharide. No conclusion could be drawn from the overall diminished antibody response observed with those prepared with rEPA or DT using similar conjugation chemistries and polysaccharide sizes (Fig. 3).

### Tetravalent conjugate (ACWY) PS:PR loading ratio

**Fig. 4:**
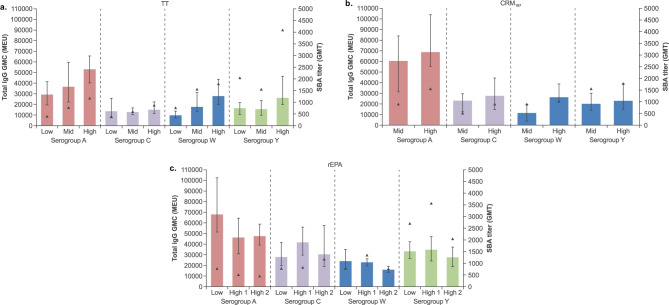
Effect of PS:PR loading ratio on immunogenicity of tetravalent (ACWY) conjugates prepared with **a** TT, **b** CRM_197_, and **c** rEPA protein carriers (0.25 µg polysaccharide dose per serogroup) via reductive amination. Individual serogroup polysaccharide loading ratios to total protein were as follows: ‘low’ (~ 0.3 to ~ 0.5), ‘mid’ (~ 0.5 to ~ 0.7) or ‘high’ (~ 0.7 or higher). See Table S2 for formulation description and key physiochemical properties. Bars represent total IgG level (GMCs with 95% CIs) and triangles represent SBA titer (GMT; sub-pools were prepared by combining two mouse sera to obtain five sub-pools per group). MEU; mouse ELISA units

The polysaccharide-specific antibody responses (total IgG and bactericidal) induced by the tetravalent (ACWY) polysaccharide conjugated formulations (prepared by reductive amination using the same polysaccharide size per serogroup, and compared at the same PS:PR loading ratio) did not differ markedly by carrier proteins (Fig. 4). In addition, there were no marked differences in antibody responses between conjugates with different PS:PR loading ratios. There was a trend, however, of increased polysaccharide-specific antibody responses (total IgG and bactericidal) with increased PS:PR loading ratio of the tetravalent (ACWY) polysaccharide TT- and CRM_197_- conjugated formulations (Fig. 4a–b), but this trend was not as apparent with the rEPA conjugated formulations (Fig. 4c).

### Effect of de-*O*-acetylation of serogroups C, W, and Y

**Fig. 5:**
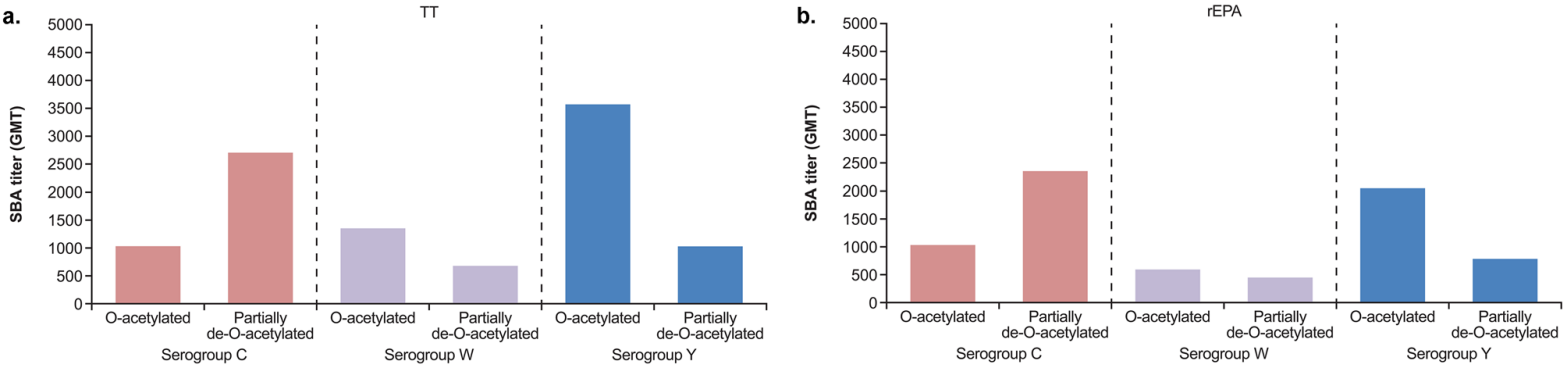
Effect on immunogenicity of partial de-*O*-acetylation of serogroups C, W, and Y conjugates prepared with **a** TT, and **b** rEPA protein carriers (0.25 µg polysaccharide dose per serogroup). Data shown for serogroup-specific bactericidal antibody levels induced by tetravalent formulations composed of *O*-acetylated serogroups ACWY (*O*-acetylated; TT or rEPA conjugated to periodate activated polysaccharide groups A [125 kDa], C [75 kDa], Y [75 kDa], W [75 kDa] via reductive amination) and *O*-acetylated serogroup A with partially de-*O*-acetylated serogroups CWY (partially de-*O*-acetylated; TT or rEPA conjugated to periodate activated polysaccharide groups A [125 kDa], partially de-*O*-acetylated C [75 kDa], partially de-*O*-acetylated Y [75 kDa], partially de-*O*-acetylated W [75 kDa] via reductive amination). SBA titers presented (GMT; sub-pools were prepared by combining two mouse sera to obtain five sub-pools per group)

The de-*O*-acetylation of serogroup A had been previously shown to have a negative impact on the bactericidal responses and thus was not pursued from the onset [16]. For the other serogroups, there were some marked differences in polysaccharide-specific bactericidal antibody responses (total IgG not reported as ELISA assay was only specific for *O*-acetylated polysaccharide antibodies) induced by the de-*O*-acetylated and *O*-acetylated polysaccharide conjugate formulations which were dependent on the serogroup (Fig. 5). Bactericidal antibody titers specific for serogroup C induced with the partially de-*O*-acetylated serogroups CWY-TT and -rEPA conjugates tended to be higher than with the respective fully *O*-acetylated conjugates. Thus, confirming the observations seen with the monovalent serogroup C conjugate described above (Fig. 3), where the partially de-*O*-acetylated serogroup C TT conjugate induced higher levels of serogroup C specific bactericidal antibodies than formulations containing fully *O*-acetylated serogroup C polysaccharide. In contrast, the de-*O*-acetylated serogroup W and Y conjugates resulted in a trend towards lower antibody responses than with the *O*-acetylated conjugates, irrespective of conjugated carrier protein. Thus, formulations containing de-*O*-acetylated serogroups W and Y were not pursued further.

### Serogroup A conjugation chemistry with and without ADH linker

Leading up to the GMP manufacturing of the monovalent conjugates for the first phase I clinical study, there was an observed inconsistency in the sodium metaperiodate activation of the serogroup A polysaccharide, and therefore, a conjugation chemistry different from that used for the other serogroups was developed for serogroup A—namely, CDI activation of the native serogroup A polysaccharide followed by direct conjugation to the protein carrier. The first phase I clinical trial showed suboptimal responses to the serogroup A conjugate that was prepared by CDI activation of the native group A polysaccharide followed by direct conjugation to the TT carrier protein. Therefore, re-development studies of the serogroup A polysaccharide conjugate were performed to improve the immunogenicity by adding ADH linkers between the CDI-activated polysaccharide and the carrier protein. This was achieved by derivatizing the CDI-activated serogroup A polysaccharide with ADH linkers, purifying the ADH-derivatized serogroup A polysaccharide, and then conjugating the ADH-derivatized polysaccharide to the carrier protein via amine coupling using EDAC. Additionally, as a back-up chemistry, the ADH/EDAC conjugation chemistry used in the preparation of Menactra (MCV4-DT; Sanofi Pasteur, Swiftwater, PA) was slightly modified by increasing the polysaccharide chain length to ~ 40 kDa (as compared to ~ 15 kDa for MCV4-DT) and conjugating it to TT via amine coupling using EDAC.

**Fig. 6:**
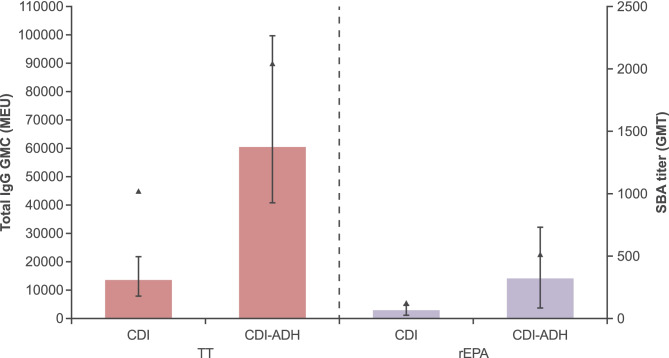
Immunogenicity of serogroup A conjugates prepared with different conjugation chemistries (with and without ADH linker) in a tetravalent formulation at 0.25 µg polysaccharide dose per serogroup (carbonyldiimidazole [CDI] activated native serogroup A polysaccharide conjugated directly to TT or rEPA; CDI-ADH, CDI-activated native serogroup A polysaccharide derivatized with ADH prior to conjugation to TT or rEPA via amine coupling using EDAC). See Table S3 for formulation description and key physiochemical properties. Bars represent total IgG level (GMCs with 95% CIs) and triangles represent SBA titer (all sera were pooled into one pool and tested)

**Fig. 7:**
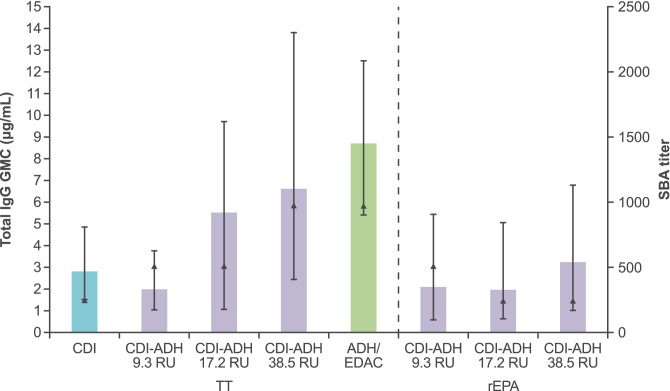
Immunogenicity associated with increased amounts of ADH linkers added to the serogroup A polysaccharide (ADH linkers added to native serogroup A CDI-activated polysaccharide, prior to conjugating to either TT or rEPA via amine coupling using EDAC). The amount of ADH linker added per serogroup A polysaccharide was defined as a mole ratio of polysaccharide repeat units (RU)/ADH linker, where the higher the number of polysaccharide RU units/ADH linker means fewer ADH linkers were added per polysaccharide chain. Also included for comparison are immunogenicity data for serogroup A TT conjugates produced by CDI-activated polysaccharide conjugated directly to TT and by ADH/EDAC (MCV4-DT) linker chemistry. See Table S4 for formulation description and key physiochemical properties. Bars represent total IgG level (GMCs with 95% CIs) and triangles represent SBA titer (all sera were pooled into one pool and tested)

In the subsequent immunogenicity studies, some of the sera had consistently high coefficients of variation in the IgG serogroup A specific antibody levels; therefore, the total serogroup A specific IgG antibody levels reported were from serum pools (equal volume of serum from each mouse in the group). Total serogroup A specific IgG antibody induced in response to tetravalent TT or rEPA conjugates were highest with formulations containing serogroup A conjugates produced using CDI-ADH/EDAC (linker) chemistry, or ADH/EDAC (MCV4-DT) linker chemistry, compared to those containing serogroup A conjugates produced using CDI chemistry (no linker) (Figs. 6 and 7). Tetravalent rEPA conjugated formulations were less immunogenic in inducing serogroup A specific IgG antibody responses than tetravalent TT conjugated formulations produced using the same conjugation chemistry.

#### Serogroup A conjugation chemistry CDI/ADH linker: effect of number of repeat units on immunogenicity

There was a trend indicating that CDI/ADH serogroup A-TT conjugate formulations having the fewest ADH linkers (38.5 polysaccharide RU/ADH linker) had the highest antibody responses among conjugates prepared using CDI conjugation chemistry (Fig. 7). This trend was not particularly evident with serogroup A-rEPA conjugates.

## Discussion

At the outset of the next generation meningococcal conjugate vaccine development, one of the first decisions made was regarding which carrier protein to use. There were several carrier proteins initially available for use at the time including TT, DT, and rEPA, and later CRM_197_. Each carrier protein had its own physiochemical characteristics that had to be taken into consideration. For example, the toxoided proteins (TT and DT) had fewer ε-amino groups available from the lysine residues on the protein surface due to the toxoiding process, which would limit the reactivity of those proteins depending on the conjugation chemistry used. The size of the different carrier proteins was also taken into consideration as this varies from ~ 60 kDa for DT and CRM_197_ [17, 18], ~ 66 kDa for rEPA [19], and ~ 150 kDa for TT [20]. The size of the carrier protein has implications not only for the overall size of the resulting conjugates, but also on the purification strategies required to remove non-covalently bound carrier protein (“free protein”) from the glycoconjugates.

One of the original goals was to develop a meningococcal conjugate vaccine that would be highly efficacious in the infants and older adults. Although included in the developmental program as a comparator, DT was de-prioritized as one of the potential carrier protein candidates because data suggested the need for improved immune responses in the youngest infants [21]. Thus, screening for another carrier protein focused on TT, CRM_197_ and rEPA.

In addition to selecting which carrier protein to use, there were several other physiochemical characteristics to consider including: conjugation chemistry; linker vs. no linker; neoglycoconjugate (polysaccharide end group linked via a single covalent attachment to carrier protein) vs. lattice (multiple attachments between several polysaccharides and carrier proteins); polysaccharide size; PS:PR loading ratio; and *O*-acetyl content. Stability of the monovalent glycoconjugates was another key consideration for selection when the different conjugation chemistries were evaluated. In our experience, the most stable glycoconjugates are large lattice-like conjugates with multipoint covalent attachments between the carrier protein and polysaccharide chains. As such, hydrolysis of the polysaccharide that occurs over time in aqueous solution is less likely to result in liberation of oligosaccharides from carrier protein/conjugate complexes and loss of vaccine potency, indicated by the level of free polysaccharide. As demonstrated by us recently, despite certain polysaccharide chains being innately less stable than others, polysaccharide chains in lattice-like conjugates with multipoint covalent attachment remain attached to the carrier protein even after a potential breakage of an attachment [22]. However, in the case of neoglycoconjugates, where the polysaccharide chains are end-linked to the carrier protein, any polysaccharide chain break results in the liberation of the polysaccharide chain from the carrier protein/conjugate complex. Additionally, the number of multipoint, covalent attachments between the carrier protein and polysaccharide chain may also have implications for the resulting immune response [23, 24]. Lastly, large size conjugates are desired for the infant population as previous data from *Haemophilus influenzae type b* conjugate development indicated that larger glycoconjugates performed better in the infant population than smaller glycoconjugates [25]*.*

Selection of a conjugation chemistry that produced large lattice conjugates, with good stability and immunogenicity, as well as ease of manufacturing were the main considerations during preclinical studies conducted to identify lead candidates. This was achieved by producing several glycoconjugates on a small scale for immunogenicity evaluation in mouse studies (presented here), in combination with physicochemical characterization and stability studies [22]. From our initial studies of serogroup C monovalent conjugates, TT emerged as one of the most effective carrier proteins. In addition, serogroup C conjugate preparations that had undergone partial de-*O*-acetylation prior to conjugation induced higher antibody responses than those that did not. In general, the size of serogroup C polysaccharide units, ranging from 10 to 50 kDa, showed improved SBA titers with increasing size; however, as the size of the polysaccharide chain increased, so did the PS:PR loading ratios and therefore it was not possible to decouple these two physiochemical attributes. In general, there were no considerable SBA titer differences among the different conjugation chemistries assessed; reductive amination and Br-Cys conjugation all produced immunogenic conjugates. However, Br-Cys conjugation was deprioritized for two reasons: 1) preference for large lattice conjugates for improved stability and potentially greater immunogenicity in infants, and 2) ease of GMP manufacturing (fewer process steps preferred). Therefore, the early developmental program focused on reductive amination conjugation chemistry, directly conjugating the activated polysaccharide to the carrier proteins without linkers.

Subsequent assessments of a series of ACWY tetravalent conjugate preparations using reductive amination showed no substantial differences between the different carrier proteins (TT, rEPA and CRM_197_); however, there was a trend of increasing immunogenicity with increased PS:PR loading ratio for TT and CRM_197_ conjugate preparations. Therefore, it was decided in general to target PS:PR loading ratios from ~ 0.6 to 1.2 for further development. In addition, the *O*-acetyl content for serogroups CWY also had an impact on immunogenicity; partial de-*O*-acetylation of serogroup C polysaccharide increased antibody responses consistent with previous observations in mice and humans [26, 27]; whereas, partial de-*O*-acetylation of serogroups W and Y polysaccharides reduced antibody responses and therefore, it was decided to not to pursue de-*O*-acetylation of serogroups W and Y any further. The de-*O*-acetylation of serogroup A had been previously shown to have negative impact on the bactericidal responses and was thus not pursued from the onset [16].

We undertook extensive efforts to select the most appropriate mouse model for assessing the immunogenicity of our meningococcal conjugate preparations that would be sufficiently able to differentiate between conjugated polysaccharides and against unconjugated free polysaccharides, as well as to degradation of vaccine preparations [13]. Nonetheless, the predictions as to the required features of the glycoconjugate to improve immunogenicity and generate a functional immune response would only be applicable to the particular polysaccharide assessed, and would also need to be confirmed in clinical studies. Indeed, mouse studies revealed that the addition of AlPO_4_ adjuvant to trivalent (CWY) or tetravalent (ACWY) rEPA and TT conjugate formulations did not improve the antibody response considerably compared to those obtained with the corresponding unadjuvanted formulations, with the exception of a single initial study (data not shown). The first human phase I clinical trial that evaluated the potential influence of AlPO_4_ adjuvant on the immunogenicity of a tetravalent (ACWY) TT conjugate formulation also did not demonstrate any considerable enhancement of antibody response with addition of the adjuvant [28]. Therefore, MenACYW-TT was further developed and licensed as a non-adjuvanted vaccine.

The mouse immunogenicity model also rightly predicted (Figs. 6 and 7) the insufficient human immune response to the serogroup A conjugate observed in the first phase I clinical trial [28]; the first serogroup A conjugate assessed in humans was formed by directly conjugating CDI-activated native serogroup A polysaccharide to TT, with no linker. Therefore, other conjugation chemistries were assessed to improve immunogenicity to serogroup A conjugates. For improved immunogenicity, the ADH linker was added to the CDI-activated polysaccharide, and then subsequently conjugated to TT via amine coupling using EDAC. Additionally, as a back-up option, the ADH/EDAC conjugation chemistry used in the preparation of MCV4-DT was slightly modified by increasing the polysaccharide chain length (to ~ 40 kDa as compared to ~ 15 kDa for MCV4-DT) and conjugating it to TT. All of the conjugates prepared with serogroup A polysaccharide chains derivatized with ADH linkers, followed by conjugation to the carrier proteins (TT or rEPA) via amine coupling using EDAC induced higher antibody responses in the mouse model than conjugates prepared using CDI-direct chemistry (no linkers) (Figs. 6 and 7). The number of ADH linkers, however, that should be added to the CDI-activated/ADH-derivatized serogroup A polysaccharide, prior to conjugation to either TT or rEPA via amine coupling using EDAC remained to be established. Serogroup A polysaccharide conjugates with the fewest ADH linkers per polysaccharide induced the highest total IgG antibody responses. It is not clear, however, if the results were biased by the animal model as prior knowledge indicated that certain animal models respond better to neoglycoprotein conjugates with polysaccharides end linked to the carrier proteins (MCV4-DT linker chemistry). Therefore, in a subsequent clinical trial, both types of serogroup A conjugates were tested in humans [28], and the results of that trial indicated that the CDI-activated/ADH-derivatized serogroup A polysaccharide conjugated to TT via amine coupling using EDAC was the better conjugate.

In summary, development of the quadrivalent meningococcal conjugate vaccine which is indicated for use in all age groups and with improved immunogenicity compared to MCV4-DT resulted from an iterative process that combined preclinical studies, with ‘fine-tuning’ process adjustments based on the immunogenicity results obtained from the early clinical trials. The immunogenicity trends observed in the mouse model informed development of the first versions of the conjugated vaccine which were then assessed in early phase I clinical trials. Accordingly, polysaccharides of > 50 kDa size conjugated to TT at a high polysaccharide to protein ratio via reductive amination for serogroups C, W and Y, and CDI/ADH chemistry for serogroup A were chosen for our new tetravalent meningococcal conjugate vaccine (MenACYW-TT). The high immunogenicity of the lead tetravalent conjugate vaccine empirically identified from the extensive preclinical and clinical exploratory studies, further enhanced by formulation at a higher antigen dose relative to its predecessor, was ultimately confirmed in clinical studies by the robust antibody responses observed across all four serogroups in all age groups including adults and the most challenging age group, infants [29]. Additional phase III studies in infants as young as 6 weeks’ old are currently on going (NCT03547271, NCT03691610, NCT03673462, NCT03630705, NCT03537508, and NCT03632720) with the potential to support use of MenACYW-TT from this very young age through to the elderly worldwide.

## Supplementary information

Below is the link to the electronic supplementary material.

Supplementary file1 (DOCX 119 KB)

## Data Availability

The data that support the findings presented in the current report are included in this article and the supplementary material.
