# Supplementary material for: Preclinical development of the quadrivalent meningococcal (ACYW) tetanus toxoid conjugate vaccine, MenQuadfi®
Source: Glycoconj J. 2022 Apr 20;39(3):381–92. doi: 10.1007/s10719-022-10050-2 (PMC9019543; doi:10.1007/s10719-022-10050-2)
Supplement: Supplementary file 1 — Supplementary file1 (DOCX 119 KB) [file 10719_2022_10050_MOESM1_ESM.docx]

**Supplementary material**

Table S1. Physicochemical characteristics of the monovalent serogroup C conjugates formulated assessment of multiple conjugation parameters.

| **Sample descriptions** | **PS:PR** | **Multiangle light scattering (MALS) (M*w*)** | ***O*-acetyl (μmoles per mg PS)** | **µmoles O-acetyl per µmole PS RU** | **% Free PS** |
| --- | --- | --- | --- | --- | --- |
| rEPA + 10kDa | 0.21 | 145,900 | 2.74 | 0.9 | 0.0 |
| TT + 10 kDa | 0.11 | 507,930 | 4.17 | 1.3 | 0.0 |
| DT + 10 kDa | 0.30 | 145,700 | 3.17 | 1.0 | 0.0 |
|  |  |  |  |  |  |
| rEPA + 20kDa | 0.21 | 150,00 | 2.43 | 0.8 | 0.0 |
| TT + 20 kDa | 0.17 | 580,720 | 2.35 | 0.9 | <10.0 |
| DT + 20 kDa | 0.40 | 168,000 | 2.73 | 0.9 | 0.0 |
|  |  |  |  |  |  |
| rEPA + 50kDa | 0.36 | 191,200 | 2.07 | 0.7 | 0.0 |
| TT + 50 kDa | 0.31 | 635,420 | 1.99 | 0.6 | 0.0 |
| DT + 50 kDa | 0.47 | 315,000 | 2.25 | 0.7 | 2.0 |
|  |  |  |  |  |  |
| rEPA + 20kDa de-acetyl | 0.26 | 138,300 | 0.86 | 0.3 | <10.0 |
| TT + 20 kDa de-acetyl | 0.23 | 430,020 | 1.14 | 0.4 | <10.0 |
|  |  |  |  |  |  |
| rEPA + 50kDa de-acetyl | 0.52 | 977,620 | 1.10 | 0.4 | 0.0 |
| TT + 50 kDa de-acetyl | 0.44 | 376,220 | 1.18 | 0.4 | 0.0 |
| DT + 50 kDa de-acetyl | 0.48 | 111,320 | 1.11 | 0.4 | 0.0 |
|  |  |  |  |  |  |
| ADH-rEPA + 10kDa | 0.12 | 175,820 | 1.95 | 0.6 | 3.3 |
| ADH-rEPA + 20kDa | 0.16 | 202,620 | 2.30 | 0.7 | <10.0 |
| ADH-rEPA + 50kDa | 0.31 | 261,120 | 1.88 | 0.6 | 5.9 |
|  |  |  |  |  |  |
| DT + ADH-GCM | 0.25 | 335,920 | 2.86 | 0.9 | 3.4 |

PR, protein; PS, polysaccharide; RU, repeat unitTable S2. Description of the monovalent conjugates and corresponding PS:PR loading ratios for the monovalent conjugates studied as tetravalent (ACWY) formulations in mice.

| **Product** | **Lot #** | **Sample description** | **Components** | **PS:PR loading ratio** |
| --- | --- | --- | --- | --- |
| Low loading rEPA | 160c-80A | rEPA conjugated via reductive amination to 75 or 125 kDa serogroups A, C, Y, W polysaccharides | 125kDa MenA:rEPA | 0.38 |
|  |  |  | 75kDa MenC:rEPA | 0.38 |
|  |  |  | 75kDa MenY:rEPA | 0.49 |
|  |  |  | 75kDa MenW:rEPA | 0.47 |
| High loading rEPA 1 | 160c-80B | rEPA conjugated via reductive amination to 75 or 125 kDa serogroups A, C, Y, W polysaccharides | 125kDa MenA:rEPA | 0.56 |
|  |  |  | 75kDa MenC:rEPA | 0.79 |
|  |  |  | 75kDa MenY:rEPA | 1.13 |
|  |  |  | 75kDa MenW:rEPA | 0.82 |
| High loading rEPA 2 | 160c-80C | rEPA conjugated via reductive amination to 75 or 125 kDa serogroups A, C, Y, W polysaccharides | 125kDa MenA:rEPA | 0.75 |
|  |  |  | 75kDa MenC:rEPA | 1.02 |
|  |  |  | 75kDa MenY:rEPA | 1.27 |
|  |  |  | 75kDa MenW:rEPA | 1.46 |
| Low loading TT | 160c-80D | TT conjugated via reductive amination to 75 or 125 kDa serogroups A, C, Y, W polysaccharides | 125kDa MenA:TT | 0.32 |
|  |  |  | 75kDa MenC:TT | 0.35 |
|  |  |  | 75kDa MenY:TT | 0.3 |
|  |  |  | 75kDa MenW:TT | 0.2 |
| Mid loading TT | 160c-80E | TT conjugated via reductive amination to 75 or 125 kDa serogroups A, C, Y, W polysaccharides | 125kDa MenA:TT | 0.52 |
|  |  |  | 75kDa MenC:TT | 0.55 |
|  |  |  | 75kDa MenY:TT | 0.54 |
|  |  |  | 75kDa MenW:TT | 0.51 |
| High loading TT | 160c-80F | TT conjugated via reductive amination to 75 or 125 kDa serogroups A, C, Y, W polysaccharides | 125kDa MenA:TT | 0.82 |
|  |  |  | 75kDa MenC:TT | 0.62 |
|  |  |  | 75kDa MenY:TT | 0.99 |
|  |  |  | 75kDa MenW:TT | 0.73 |
| Mid loading CRM_197_ | 160c-80G | CRM_197_ conjugated via reductive amination to 75 kDa serogroups A, C, Y, W polysaccharides | 75kDa MenA:CRM_197_ | 0.54 |
|  |  |  | 75kDa MenC:CRM_197_ | 0.54 |
|  |  |  | 75kDa MenY:CRM_197_ | 0.74 |
|  |  |  | 75kDa MenW:CRM_197_ | 0.52 |
| High loading CRM_197_ | 160c-80H | CRM_197_ conjugated via reductive amination to 75 or 125 kDa serogroups A, C, Y, W polysaccharides | 125kDa MenA:CRM_197_ | 0.73 |
|  |  |  | 75kDa MenC:CRM_197_ | 1.34 |
|  |  |  | 75kDa MenY:CRM_197_ | 1.45 |
|  |  |  | 75kDa MenW:CRM_197_ | 1.24 |

Table S3. Physicochemical characteristics of the monovalent conjugates formulated as tetravalent conjugates for serogroup A conjugation chemistry comparison.

| **Sample description** | **Monovalent Lot #** | **PS:PR** | ***O*-acetyl (µmoles per mg PS)** | **µmoles O-acetyl per µmole PS RU** | **% Free PS** |
| --- | --- | --- | --- | --- | --- |
| **Tetravalent Lot # 160c-173A** |  |  |  |  |  |
| rEPA-ADH/EDAC GAM | 160c-141A | 0.72 | 2.00 | 0.6 |  |
| rEPA-GCM | 171c-038 | 0.98 | 2.95 | 0.9 | 1.5% |
| rEPA-GYM | 171c-037 | 1.05 | 1.14 | 0.6 | 3.2% |
| rEPA-GWM | 160c-120B | 1.03 | 1.46 | 0.7 | 4.8% |
| **Tetravalent Lot # 160c-173C** |  |  |  |  |  |
| rEPA-CDI GAM | 160c-172c | 1.05 | 1.92 | 0.6 |  |
| rEPA-GCM | 171c-038 | 0.98 | 2.95 | 0.9 | 1.5% |
| rEPA-GYM | 171c-037 | 1.05 | 1.14 | 0.6 | 3.2% |
| rEPA-GWM | 160c-120B | 1.03 | 1.46 | 0.7 | 4.8% |
| **Tetravalent Lot # 160c-173E** |  |  |  |  |  |
| TT-ADH/EDAC GAM | 160c-141C | 0.62 | 3.310 | 1.0 |  |
| TT-GCM | UD06610 | 0.60 | 2.40 | 0.8 | < 3.0% |
| TT-GYM | UD06612 | 0.96 | 1.30 | 0.6 | < 3.0% |
| TT-GWM | UD06611 | 0.92 | 1.60 | 0.8 | 4.0% |
| **Tetravalent Lot # 160c-173F** |  |  |  |  |  |
| TT-CDI GAM | 160c-172e | 1.20 | 1.62 | 0.5 |  |
| TT-GCM | UD06610 | 0.60 | 2.40 | 0.8 | < 3.0% |
| TT-GYM | UD06612 | 0.96 | 1.30 | 0.6 | < 3.0% |
| TT-GWM | UD06611 | 0.92 | 1.60 | 0.8 | 4.0% |

PR, protein; PS, polysaccharide; RU, repeat unit

Table S4. Physicochemical characteristic testing results for the monovalent conjugates formulated as tetravalent conjugates for serogroup A conjugation chemistries comparison*.*

| **Sample descriptions** | **Lot #** | **PS:PR** | **MALLS (M*w*)** | **Kd** | ***O*-acetyl (μmoles per mg PS)** | **µmoles O-acetyl per µmole PS RU** | **% Free PS** |
| --- | --- | --- | --- | --- | --- | --- | --- |
| **Phase I/II GMP Lot** |  |  |  |  |  |  |  |
| TT CDI GAM | UD07987 | 0.63 | 1.29E+06 | 0.31 | 2.3 | 0.7 | <4 |
| TT GCM | UD07973 | 0.53 | 1.04E+06 | 0.34 | 2.4 | 0.8 | 5% |
| TT GYM | UD07975 | 0.85 | 1.08E+06 | 0.34 | 1.2 | 0.6 | 11% |
| TT GWM | UD07974 | 0.88 | 1.11E+06 | 0.32 | 1.3 | 0.6 | <3% |
| **TT CDI/ADH GAM (1 ADH per 9.3 repeat units RU)** |  |  |  |  |  |  |  |
| TT CDI/ADH GAM | 840-041B | 1.48 | 1,516,000 | 0.21 |  |  | <5% |
| TT GCM | UD07973 | 0.53 | 1.04E+06 | 0.34 | 2.4 | 0.8 | 5% |
| TT GYM | UD07975 | 0.85 | 1.08E+06 | 0.34 | 1.2 | 0.6 | 11% |
| TT GWM | UD07974 | 0.88 | 1.11E+06 | 0.32 | 1.3 | 0.6 | <3% |
| **TT CDI/ADH GAM (1 ADH per 17.2 repeat units RU)** |  |  |  |  |  |  |  |
| TT CDI/ADH GAM | 840-048A | 1.21 | 1,661,000 | 0.23 |  |  | 0% |
| TT GCM | UD07973 | 0.53 | 1.04E+06 | 0.34 | 2.4 | 0.8 | 5% |
| TT GYM | UD07975 | 0.85 | 1.08E+06 | 0.34 | 1.2 | 0.6 | 11% |
| TT GWM | UD07974 | 0.88 | 1.11E+06 | 0.32 | 1.3 | 0.6 | <3% |
| **TT CDI/ADH GAM (1 ADH per 38.5 repeat units RU)** |  |  |  |  |  |  |  |
| TT CDI/ADH GAM | 840-048E | 1.20 | 1,505,000 | 0.25 |  |  |  |
| TT GCM | UD07973 | 0.53 | 1.04E+06 | 0.34 | 2.4 | 0.8 | 5% |
| TT GYM | UD07975 | 0.85 | 1.08E+06 | 0.34 | 1.2 | 0.6 | 11% |
| TT GWM | UD07974 | 0.88 | 1.11E+06 | 0.32 | 1.3 | 0.6 | <3% |
| **rEPA CDI/ADH GAM (1 ADH per 9.3 repeat units RU)** |  |  |  |  |  |  |  |
| rEPA-CDI/ADH GAM | 840-041A | 1.31 | 1,192,000 | 0.20 |  |  | 0% |
| rEPA-GCM | UD06911 | 0.90 | 357,800 | 0.46 | 2.30 | 0.7 | 0.8% |
| rEPA-GYM | UD06913 | 1.28 | 654,300 | 0.35 | 1.20 | 0.6 | 1.8% |
| rEPA-GWM | UD06912 | 1.30 | 670,100 | 0.33 | 1.60 | 0.8 | 6.0% |
| **rEPA CDI/ADH GAM (1 ADH per 17.2 repeat units RU)** |  |  |  |  |  |  |  |
| rEPA-CDI/ADH GAM | 840-048C | 1.30 | 1,047,000 | 0.22 |  |  | 0% |
| rEPA-GCM | UD06911 | 0.90 | 357,800 | 0.46 | 2.30 | 0.7 | 0.8% |
| rEPA-GYM | UD06913 | 1.28 | 654,300 | 0.35 | 1.20 | 0.6 | 1.8% |
| rEPA-GWM | UD06912 | 1.30 | 670,100 | 0.33 | 1.60 | 0.8 | 6.0% |
| **rEPA CDI/ADH GAM (1 ADH per 38.5 repeat units RU)** |  |  |  |  |  |  |  |
| rEPA-CDI/ADH GAM | 840-048G | 1.37 | 891,600 | 0.24 |  |  |  |
| rEPA-GCM | UD06911 | 0.90 | 357,800 | 0.46 | 2.30 | 0.7 | 0.8% |
| rEPA-GYM | UD06913 | 1.28 | 654,300 | 0.35 | 1.20 | 0.6 | 1.8% |
| rEPA-GWM | UD06912 | 1.30 | 670,100 | 0.33 | 1.60 | 0.8 | 6.0% |
| **TT ADH/EDAC (MCV4-DT) linker chemistry** |  |  |  |  |  |  |  |
| TT ADH GAM | 160c-198 | 0.51 | 1.70E+06 |  | 2.9 | 0.9 | 9.7% |
| TT GCM | UD07973 | 0.53 | 1.04E+06 | 0.34 | 2.4 | 0.8 | 5% |
| TT GYM | UD07975 | 0.85 | 1.08E+06 | 0.34 | 1.2 | 0.6 | 11% |
| TT GWM | UD07974 | 0.88 | 1.11E+06 | 0.32 | 1.3 | 0.6 | <3% |

PR, protein; PS, polysaccharide; RU, repeat unitFigure S1. Conjugation chemistries assessed: a) reductive amination; b) CDI activation, CDI activation/ADH derivatization; c) EDAC conjugation; and d) thioether conjugation by cystamine derivatization of polysaccharide and bromination of the carrier proteins

a) reductive amination

b) CDI activation of polysaccharide hydroxyls/CDI activation of polysaccharide hydroxyls followed by derivatization with ADH

c) EDAC mediated amine coupling

d) thioether conjugation

Adapted from Hermanson GT. Bioconjugate Techniques 3rd Edition 2013. Academic Press
